# Urinary non-albumin protein-creatinine ratio is an independent predictor of mortality in patients with type 2 diabetes: a retrospective cohort study

**DOI:** 10.1038/s41598-024-61395-7

**Published:** 2024-05-08

**Authors:** Yu-Cheng Cheng, Chin-Li Lu, Chiann-Yi Hsu, Meei-Ling Sheu, I-Te Lee

**Affiliations:** 1 Division of Endocrinology and Metabolism, Department of Internal Medicine, Taichung Veterans General Hospital, No. 1650, Section 4, Taiwan Boulevard, Taichung, 40705 Taiwan; 2 School of Medicine, National Yang Ming Chiao Tung University, Taipei, 11221 Taiwan; 3 Institute of Biomedical Sciences, National Chung Hsing University, Taichung, 40227 Taiwan; 4 Graduate Institute of Food Safety, College of Agriculture and Natural Resources, National Chung Hsing University, Taichung, 40227 Taiwan; 5 Biostatistics Task Force of Taichung Veterans General Hospital, Taichung, 40705 Taiwan; 6 School of Medicine, Chung Shan Medical University, Taichung, 40201 Taiwan

**Keywords:** Diabetes, Urinary albumin-to-creatinine ratio, Non-albumin proteinuria, Urinary non-albumin protein-creatinine ratio, Mortality, Type 2 diabetes, Diabetes complications

## Abstract

Albuminuria is a well-known predictor of chronic kidney disease in patients with type 2 diabetes mellitus (DM). However, proteinuria is associated with chronic complications in patients without albuminuria. In this retrospective cohort study, we explored whether non-albumin proteinuria is associated with all-cause mortality and compared the effects of non-albumin proteinuria on all-cause mortality between patients with and without albuminuria. We retrospectively collected data from patients with type 2 DM for whom we had obtained measurements of both urinary albumin-to-creatinine ratio (UACR) and urinary protein-to-creatinine ratio (UPCR) from the same spot urine specimen. Urinary non-albumin protein-creatinine ratio (UNAPCR) was defined as UPCR–UACR. Of the 1809 enrolled subjects, 695 (38.4%) patients died over a median follow-up of 6.4 years. The cohort was separated into four subgroups according to UACR (30 mg/g) and UNAPCR (120 mg/g) to examine whether these indices are associated with all-cause mortality. Compared with the low UACR and low UNAPCR subgroup as the reference group, multivariable Cox regression analyses indicated no significant difference in mortality in the high UACR and low UNAPCR subgroup (hazard ratio [HR] 1.189, 95% confidence interval [CI] 0.889–1.589, P = 0.243), but mortality risks were significantly higher in the low UACR and high UNAPCR subgroup (HR 2.204, 95% CI 1.448–3.356, P < 0.001) and in the high UACR with high UNAPCR subgroup (HR 1.796, 95% CI 1.451–2.221, P < 0.001). In the multivariable Cox regression model with inclusion of both UACR and UNAPCR, UNAPCR ≥ 120 mg/g was significantly associated with an increased mortality risk (HR 1.655, 95% CI 1.324–2.070, P < 0.001), but UACR ≥ 30 mg/g was not significantly associated with mortality risk (HR 1.046, 95% CI 0.820–1.334, P = 0.717). In conclusion, UNAPCR is an independent predictor of all-cause mortality in patients with type 2 DM.

## Introduction

Diabetes mellitus (DM) is one of the major causes of reduced life expectancy worldwide^1^. The global prevalence of DM among adults has risen over the past two decades and is expected to increase to 12.2% by 2045^2^. Type 2 DM accounts for over 90% of all diabetics, is the major cause of end-stage renal disease (ESRD), and is associated with high cardiovascular risk and mortality^3–5^.

Albuminuria is predictive of cardiovascular disease (CVD), progression of diabetic kidney disease (DKD), and all-cause mortality in patients with type 2 DM^6,7^. In clinical practice, albuminuria can be assessed using the urinary albumin-to-creatinine ratio (UACR) obtained from a spot urine sample. UACR is widely recommended as a marker to quantify albuminuria for DKD diagnosis and the risk assessment of diabetes-related adverse outcomes^8,9^. However, several studies recently reported that DKD may progress without presenting significant albuminuria^10–13^. Based on histological findings, typical diabetic glomerulopathy has been found in normoalbuminuric patients with type 2 DM^14,15^. Among patients with biopsy-proven diabetic glomerulopathy, renal function may decline independent of the change in the UACR value^15^. The prevalence of normoalbuminuric renal insufficiency in patients with DM has currently increased and become a prevailing phenotype of DKD^16^. Increasing mortality risk has been reported in patients with type 2 DM with normoalbuminuric renal insufficiency^17,18^. Therefore, more effective markers are required for early assessment of the mortality risk in patients with type 2 DM.

Proteinuria is composed of albuminuria and non-albumin proteinuria. Based on the pathophysiology, albuminuria typically indicates glomerular damage^19^. Non-albumin proteinuria may be primarily associated with tubulointerstitial pathology, and a previous study indicated that a low urinary albumin-protein ratio was highly associated with tubulointerstitial damage based on renal biopsies^20^. In addition to glomerular damage, tubulointerstitial damage is a major feature of diabetic nephropathy^21^. Urinary non-albumin protein-creatinine ratio (UNAPCR) is predictive of DKD progression in patients with type 2 DM^22,23^. A composite of non-albumin proteinuria and albuminuria may improve the prediction of DKD progression in patients with type 2 DM^24^. In a population consisting of 32.1% patients with DM, UNAPCR had the most linear dose–response relationship with all-cause mortality and significantly improved the risk assessment for mortality compared to UACR and urine protein-to-creatinine ratio (UPCR)^25^.

Although growing evidence suggests that non-albumin proteinuria can predict DKD progression, studies exploring the predictive value of non-albumin proteinuria on all-cause mortality in patients with type 2 DM are lacking. The present study aimed to explore whether UNAPCR is predictive of all-cause mortality in patients with type 2 DM and to compare the effects of UNAPCR on predicting all-cause mortality between patients with and without albuminuria.

## Methods

### Study population

This retrospective cohort study was conducted at the Taichung Veterans General Hospital (VGH). Patients with DM are encouraged to participate in the diabetes pay-for-payment (P4P) program in Taiwan. Patients receive regular outpatient assessments every three months and an annual comprehensive assessment that includes UACR measurement in the diabetes P4P program^26^. Moreover, the National Health Insurance Administration launched a pre-ESRD P4P program in 2006 and an early chronic kidney disease (CKD) P4P program in 2011 to deliver adequate care for patients with proteinuria or CKD. UPCR is a screening marker and is considered one of the inclusion indicators in these countrywide P4P programs for the patients with a risk of CKD progression^27,28^.

**Figure 1:**
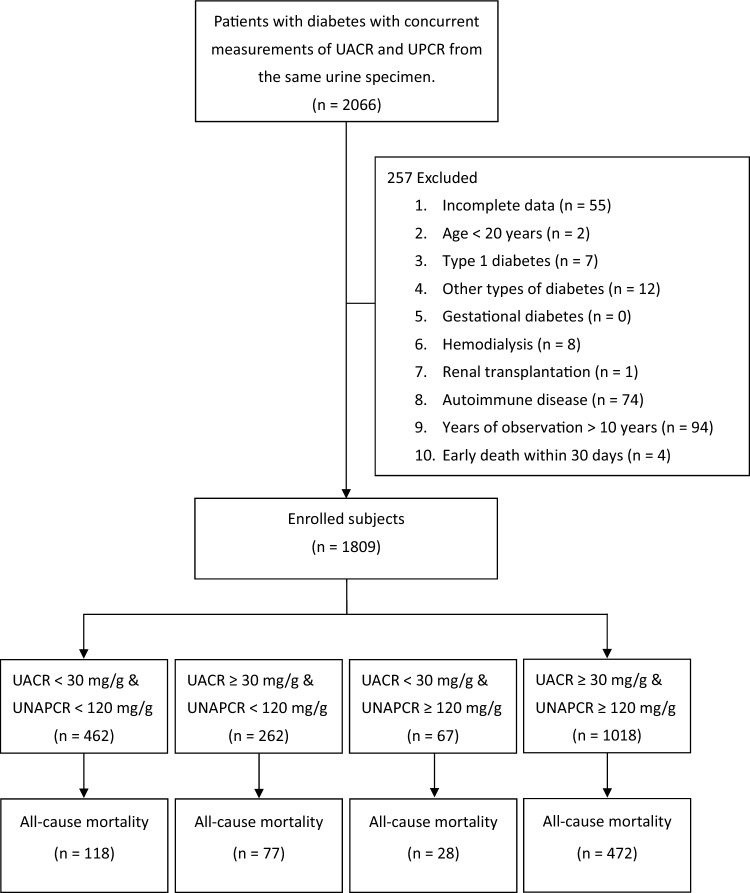
Flow diagram for the enrollment of study subjects. *UACR* urine albumin-creatinine ratio, *UNAPCR* urine non-albumin protein-creatinine ratio, *UPCR* urine protein-to-creatinine ratio.

The present study cohort enrolled patients in the diabetes P4P program between March 2008 and July 2017. The inclusion criteria were as follows: (1) outpatients with DM and (2) at least one concurrent measurement of UACR and UPCR from the same urine specimen. The index date was defined as the date on measuring urine specimen (multiple measurements were reported, the earliest data was recorded). The exclusion criteria were as follows: (1) incomplete data of other baseline characteristics; (2) age < 20 years; (3) type 1 DM; (4) other types of DM, including pancreatic, hepatic, and secondary to endocrine diseases; (5) gestational diabetes; (6) history of hemodialysis at or before the index date; (7) history of renal transplantation at or before the index date; (8) history of autoimmune disease; (9) patients with > 10 years of observation; and (10) early death within 30 days after the index date (Fig. 1).

Anonymous demographic characteristics and laboratory data were obtained from the Clinical Informatics Research and Development Center of Taichung VGH after delinking the identification information. The study protocol was approved by the Institutional Review Board of Taichung VGH in Taiwan with a waiver for obtaining informed consent. All methods were performed in accordance with the relevant guidelines and regulations.

### Endpoint and follow-up

The primary endpoint of the present study was death from any cause. Information on deaths was obtained from the Death Registry conducted by the Ministry of Health and Welfare, Executive Yuan, Taiwan, and updated by December 31, 2022. All enrolled patients were followed from the index date until death or December 31, 2022.

### Measurements

Data from the same sample of spot urine, including the total proteinuria, albuminuria, and creatinine values, were collected from each patient. UACR and UPCR were calculated as the ratio of urine albumin and protein to creatinine (mg/g), respectively. We estimated the amount of urinary non-albumin proteinuria through the following calculation: UNAPCR = UPCR − UACR. Increased albuminuria was defined as UACR ≥ 30 mg/g^8^, and increased UNAPCR was defined as UNAPCR ≥ 120 mg/g^24^. Urinary creatinine levels were determined using the Jaffé method (Advia 1800; Siemens). Urinary protein levels were determined using the dye-binding assay, and urinary albumin levels were assessed using the immune-turbidimetric method (Advia 1800; Siemens).

The other baseline characteristics, including age, sex, height, body weight, systolic blood pressure (SBP), diastolic blood pressure (DBP), glycated hemoglobulin (HbA1c), fasting plasma glucose, serum levels of creatinine, total cholesterol, high-density lipoprotein (HDL) cholesterol, low-density lipoprotein (LDL) cholesterol, triglycerides (TG), glutamic pyruvic transaminase (GPT), hemoglobulin (Hb), were retrieved at or within six months before the index date. Among multiple measurements during the baseline period, only the latest data were collected. The use of antidiabetic, antihypertensive, and lipid-lowering drugs was defined as medications prescribed within six months before the index date.

In DM management clinical practice, blood samples for biochemical analyses were collected in the morning after the patient fasted overnight. HbA1c levels were measured using cation-exchange high-performance liquid chromatography (National Glycohemoglobin Standardization Program, G8, TOSOH, Tokyo, Japan). Biochemical analyses were performed using a photometric enzymatic method with a chemical analyzer (Hitachi 7600, Tokyo, Japan). Body mass index (BMI) was calculated as the weight (kg)/(height [m])^2^. The estimated glomerular filtration rate (eGFR) was calculated using the modification of diet in renal disease equation: 186 × (serum creatinine)^−1.154^ × (age)^−0.203^ (× 0.742 if female)^29^.

Diseases were defined according to the Diagnostic International Classification of Diseases (ICD) code for the ICD 9th version or ICD 10th version. The ICD codes used in this study are shown in the Additional file 1: Table S1. A disease was identified if one patient had been diagnosed at least twice at an outpatient visit. Hypertension was defined as the use of any antihypertensive drugs within six months prior to the index date or with the diagnosis of hypertension. CKD was defined as an eGFR < 60 mL/min/1.73 m^2^. Hypercholesterolemia was defined as an LDL level ≥ 100 mg/dL, and hypertriglyceridemia was defined as a TG level ≥ 150 mg/dL according to the reference target goal^9^. CVD was defined as at least one diagnosis of coronary arterial disease, cerebral vascular disease, and/or peripheral arterial disease. Autoimmune disease was defined as at least one diagnosis of systemic lupus erythematosus, rheumatoid arthritis, sicca syndrome, and/or anti-neutrophil cytoplasmic autoantibody (ANCA)-associated vasculitis.

### Statistical analysis

Continuous variables were summarized as the mean and standard deviation. A Mann–Whitney U-test was conducted to detect significant differences in continuous variables between the two groups. A Kruskal–Wallis test was used to detect significant differences in continuous variables among more than two groups. Chi-squared tests were conducted to detect differences in categorical variables. Pearson’s correlation coefficient (*r*) was employed to determine the correlation between UACR and UNAPCR. The cumulative risk of all-cause mortality was estimated using the Kaplan–Meier method and compared using the log-rank test across groups. A univariable Cox proportional hazard regression model was used to identify potential risk factors. A multivariable Cox proportional hazards regression analysis was conducted to identify independent risk factors of mortality, and hazard ratio (HR) and 95% confidence interval (CI) were calculated. The effect of the interaction between dichotomized UACR and UNAPCR on mortality was examined in the multivariable Cox model in which dichotomized UACR and UNAPCR were concurrently entered, and the cross-product term was additionally included and tested. Moreover, multivariable stratified analyses were conducted to evaluate the all-cause mortality in the patient subgroups categorized by UACR and UNAPCR. A two-sided P value < 0.05 was considered statistically significant. Statistical analysis was performed using SPSS v22.0 (IBM Corp., Armonk, NY, USA).

### Ethics approval and consent to participate

The study was approved by the Institutional Review Board of Taichung Veterans General Hospital, and the need for informed consent was waived.

## Results

A total of 1809 subjects were enrolled in the study. To understand the influence of UACR and UNAPCR, we showed baseline characteristics of all subjects that were grouped based on the UACR value of 30 mg/g and the UNAPCR of 120 mg/g as shown in the Additional file 1: Table S2. A significantly higher UNAPCR was observed in subjects with a high UACR than in those with a low UACR (744.2 ± 1482.2 versus 120.8 ± 521.5 mg/g, P < 0.001). A significantly higher UACR was observed in subjects with a high UNAPCR than in those with a low UNAPCR (1245.2 ± 1824.5 versus 51.8 ± 164.6 mg/g, P < 0.001). A significant positive correlation between UACR and UNAPCR levels (*r* = 0.547, P < 0.001) was noted.

Over a median follow-up of 6.4 years (interquartile range: 4.8–7.6 years), 695 (38.4%) of the 1809 enrolled patients died. The mortality rate was 4.3 deaths/100 person-years in the low UACR group and 7.3 deaths/100 person-years in the high UACR group. The mortality rate was 4.1 deaths/100 person-years in the low UNPACR group and 8.2 deaths/100 person-years in the high UNAPCR group. The survival rates were significantly different between the low UACR and high UACR groups (P < 0.001), as well as between the low UNAPCR and high UNAPCR groups (P < 0.001; Additional file 1: Fig. S1). Multivariable Cox regression analyses (without UNAPCR adjusted) showed that a significantly higher risk for all-cause mortality was observed in subjects with a high UACR than in subjects with a low UACR (HR = 1.464, 95% CI: 1.210–1.772; P < 0.001). Multivariable Cox regression analyses (without adjusted UACR) showed that a significantly higher risk for mortality was observed in subjects with a high UNPACR than in subjects with a low UNPACR (HR = 1.699, 95% CI: 1.425–2.024; P < 0.001; Additional file 1: Table S3). Furthermore, a quartile analysis for the association between UNAPCR and all-cause mortality was conducted. The survival rates were significantly different across all groups of the quartile UNAPCR (overall log-rank test P < 0.001; Additional file 1: Fig. S2). Multivariable Cox regression analyses showed that compared to the reference group (Q1), higher mortality was observed in the Q2 (HR 1.636, 95% CI 1.272–2.105; P < 0.001), Q3 (HR 1.816, 95% CI 1.408–2.341; P < 0.001), and Q4 (HR 2.800, 95% CI 2.152–3.643; P < 0.001) groups (Additional file 1: Table S4).

The above results indicated that both UACR and UNAPCR were variables associated with all-cause mortality, and there was a positive correlation between UACR and UNAPCR. To clarify the influence of UACR and UNAPCR in concordance or disconcordance, all subjects were divided into four subgroups based on cutoff points of UACR (30 mg/g) and UNAPCR (120 mg/g). Four subgroups were formed: (1) patients with low UACR and low UNAPCR (n = 462); (2) patients with low UACR and high UNAPCR (n = 67); (3) patients with high UACR and low UNAPCR (n = 262); and (4) patients with high UACR and high UNAPCR (n = 1018) as shown in Fig. 1. There were 1480 (81.8%) patients in the concordant UACR and UNAPCR categories (low/low or high/high), and 329 (18.2%) patients in the disconcordant UACR and UNAPCR categories.

**Figure 2:**
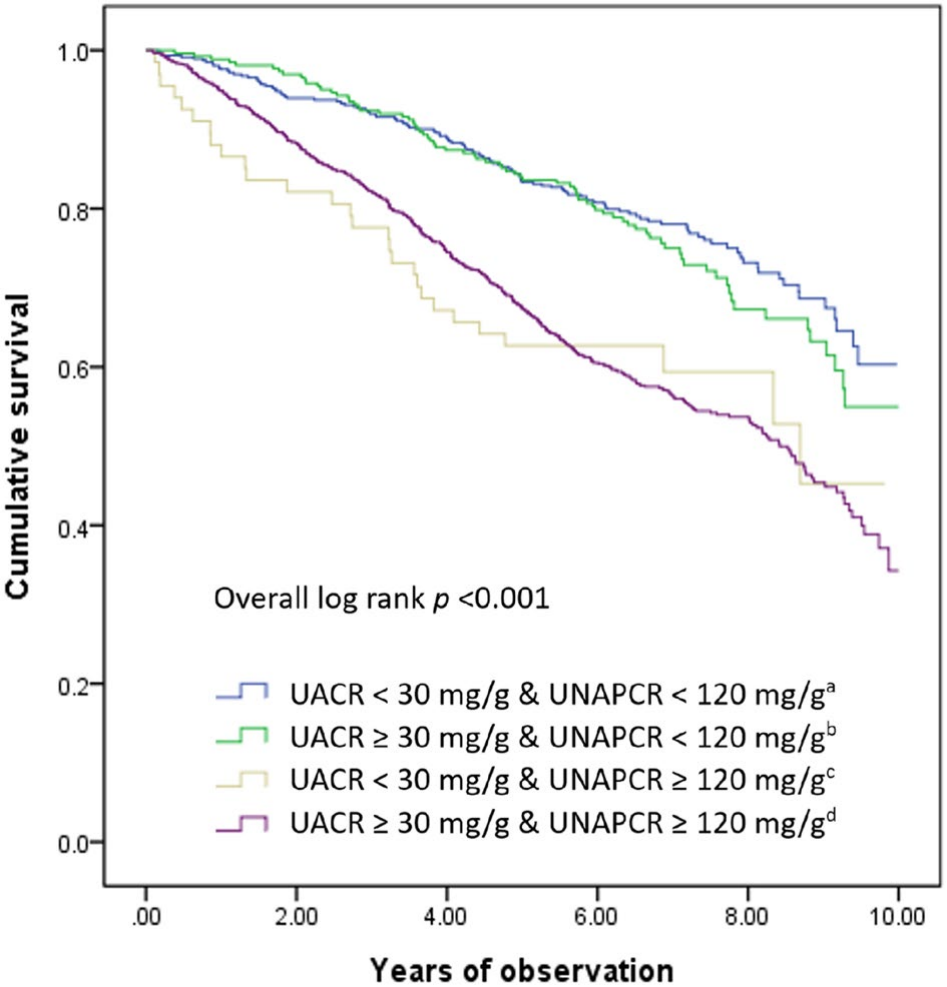
Cumulative survival curves using Kaplan–Meier method and compared by log-rank test in four groups classified by UACR and UNAPCR. Results from pairwise comparisons were as follows: a vs. b, P = 0.364; c versus d, P = 0.865; a versus c, P < 0.001; a versus d, P < 0.001; b versus c, P = 0.004; b versus d, P < 0.001. *UACR* urine albumin-to-creatinine ratio, *UNAPCR* urine non-albumin protein-creatinine ratio.

Table 1 shows the baseline characteristics of the four subgroups. These subgroups were significantly different in terms of UACR, UNAPCR, UPCR, height, weight, BMI, SBP, DBP, HbA1c, glucose, eGFR, HDL cholesterol, TG, GPT, Hb, the proportions of female patients, patients with hypertension, cancer, and glomerular disease, and patients using dipeptidyl peptidase-4 (DPP-4) inhibitors, renin-angiotensin system (RAS) blockade, hypertensive drugs, and insulin. There was no significant difference in terms of age, total and LDL cholesterol levels, and the proportions of patients with tubulointerstitial nephritis, oral hypoglycemic agent use, statin use, fibrate use, sodium–glucose cotransporter 2 (SGLT2) inhibitor use, and glucagon-like peptide-1 receptor agonist (GLP-1 RA) use among these four subgroups. The mortality rate was 3.9 deaths/100 person-years in the low UACR and low UNAPCR subgroup, 7.9 deaths/100 person-years in the low UACR and high UNAPCR subgroup, 4.4 deaths/100 person-years in the high UACR and low UNAPCR subgroup, and 8.3 deaths/100 person-years in the high UACR and high UNAPCR subgroup. The cumulative survival curves were significantly different across these four subgroups (overall log-rank test P < 0.001; Fig. 2). Pairwise comparisons show that the survival rate in the low UACR and low UNAPCR subgroup did not differ from that in the high UACR and low UNAPCR subgroup (log-rank test P = 0.364), and the survival rate in the low UACR and high UNAPCR subgroup did not differ from that in the high UACR and high UNAPCR subgroup (log-rank test P = 0.865). All other pairwise comparisons showed significant differences.

**Table 1:** Characteristics of study cohort classified into four groups based on UACR and UNAPCR levels.

	UACR < 30 mg/g (n = 529)		UACR ≥ 30 mg/g (n = 1280)		*p* value
	UNAPCR < 120 mg/g (n = 462)	UNAPCR ≥ 120 mg/g (n = 67)	UNAPCR < 120 mg/g (n = 262)	UNAPCR ≥ 120 mg/g (n = 1018)	
Age (year)	68 ± 12	71 ± 13	68 ± 13	69 ± 12	0.254
Female, n (%)	308 (66.7%)	29 (43.3%)	189 (72.1%)	606 (59.5%)	< 0.001
UACR (mg/g)	11.0 ± 7.7	17.5 ± 7.1	123.7 ± 258.5	1326.0 ± 1855.3	< 0.001
UNAPCR (mg/g)	70.1 ± 23.4	471.0 ± 1424.6	90.0 ± 20.7	912.6 ± 1619.9	< 0.001
UPCR (mg/g)	81.1 ± 26.7	488.5 ± 1424.6	213.7 ± 257.8	2238.7 ± 3012.0	< 0.001
BMI (kg/m^2^)	24.0 ± 3.7	22.8 ± 3.4	24.5 ± 3.7	23.8 ± 4.0	< 0.001
SBP (mmHg)	129 ± 17	127 ± 18	134 ± 17	137 ± 20	< 0.001
DBP (mmHg)	73 ± 12	71 ± 11	75 ± 13	75 ± 12	0.005
HbA1c (%)	7.1 ± 1.5	7.7 ± 2.2	7.4 ± 1.5	7.7 ± 1.8	0.007
Glucose (mmol/L)	8.0 ± 5.0	8.6 ± 3.8	8.1 ± 3.5	8.6 ± 4.9	< 0.001
eGFR (mL/min/1.73 m^2^)	66.7 ± 27.5	77.8 ± 49.8	65.3 ± 30.3	45.4 ± 30.4	< 0.001
Total cholesterol (mmol/L)	4.2 ± 0.9	4.3 ± 1.2	4.1 ± 0.9	4.4 ± 1.2	0.062
HDL cholesterol (mmol/L)	1.3 ± 0.4	1.2 ± 0.4	1.2 ± 0.4	1.3 ± 0.4	0.001
LDL cholesterol (mmol/L)	2.4 ± 0.8	2.4 ± 1.0	2.4 ± 0.7	2.6 ± 1.0	0.053
Triglycerides (mmol/L)	1.7 ± 1.4	1.7 ± 1.4	1.9 ± 2.1	1.9 ± 1.5	< 0.001
GPT (U/L)	30.4 ± 26.8	26.4 ± 38.2	29.6 ± 31.2	25.8 ± 22.2	< 0.001
Hb (g/L)	131 ± 18	120 ± 19	128 ± 19	116 ± 21	< 0.001
Disease, n (%)					
Hypertension, n (%)	399 (86.4%)	59 (88.1%)	246 (93.9%)	967 (95.0%)	< 0.001
CVD, n (%)	383 (82.9%)	59 (88.1%)	229 (87.4%)	897 (88.1%)	0.051
Cancer, n (%)	135 (29.2%)	29 (43.3%)	62 (23.7%)	312 (30.6%)	0.012
Glomerular disease, n (%)	57 (12.3%)	6 (9.0%)	33 (12.6%)	198 (19.4%)	< 0.001
TIN, n (%)	37 (8.0%)	3 (4.5%)	21 (8.0%)	93 (9.1%)	0.554
Drug use, n (%)					
OHA use, n (%)	379 (82.0%)	58 (86.6%)	213 (81.3%)	793 (77.9%)	0.091
Statin use, n (%)	258 (55.8%)	39 (58.2%)	163 (62.2%)	598 (58.7%)	0.414
Fibrate use, n (%)	27 (5.8%)	3 (4.5%)	24 (9.2%)	64 (6.3%)	0.274
SGLT2 Inhibitor use, n (%)	5 (1.1%)	1 (1.5%)	1 (0.4%)	2 (0.2%)	0.091
GLP-1 RA use, n (%)	4 (0.9%)	0 (0.0%)	3 (1.1%)	12 (1.2%)	0.790
DPP-4 Inhibitor use, n (%)	191 (41.3%)	40 (59.7%)	125 (47.7%)	493 (48.4%)	0.011
RAS blockade use, n (%)	221 (47.8%)	32 (47.8%)	159 (60.7%)	556 (54.6%)	0.005
Hypertensive drug use, n (%)	313 (67.7%)	40 (59.7%)	206 (78.6%)	825 (81.0%)	< 0.001
Insulin use, n (%)	88 (19.0%)	13 (19.4%)	60 (22.9%)	345 (33.9%)	< 0.001
Incidence of mortality (deaths/100 person-years)	3.9	7.9	4.4	8.3	< 0.001

Continuous data are expressed as means ± standard deviations, and categorical data are expressed as numbers (percentages).

*BMI* body mass index, *CVD* cardiovascular disease, *DBP* diastolic blood pressure, *DPP-4* dipeptidyl peptidase-4, *eGFR* estimated glomerular filtration rate, *GLP-1 RA* glucagon-like peptide-1 receptor agonist, *GPT* glutamate pyruvate transaminase, *Hb* hemoglobin, *HbA1c* hemoglobin A1c, *HDL* high-density lipoprotein, *LDL* low-density lipoprotein, *OHA* oral hypoglycemic agent, *RAS* renin-angiotensin system, *SBP* systolic blood pressure, *SGLT2* sodium–glucose cotransporter 2, *TIN* Tubulointerstitial nephritis, *UACR* urine albumin-to-creatinine ratio, *UNAPCR* urine non-albumin protein-creatinine ratio, *UPCR* urine protein-creatinine ratio.

Multivariable Cox regression analyses were performed using the low UACR and low UNAPCR subgroup as the reference group (Table 2). There was no significant difference in mortality in the high UACR and low UNAPCR subgroup compared to the reference group (HR 1.189, 95% CI 0.889–1.589; P = 0.243). Higher mortality risks were observed in the low UACR and high UNAPCR subgroup (HR 2.204, 95% CI 1.448–3.356; P < 0.001) and in the high UACR and high UNAPCR subgroup (HR 1.796, 95% CI 1.451–2.221, P < 0.001) than the reference group. Table 3 shows the analyses for the association of UACR (or UNAPCR) with all-cause mortality using Cox regression analyses stratified by UNAPCR (or UACR) levels. In the low UACR group, a significantly higher risk of mortality was observed in subjects with high UNAPCR than in subjects with low UNAPCR (HR 2.292, 95% CI 1.440–3.649; P < 0.001). In the high UACR group, a significantly higher risk of mortality was also observed in subjects with high UNAPCR than in subjects with low UNAPCR (HR 1.521, 95% CI 1.188–1.947; P = 0.001). In the low UNAPCR group, there was no significant difference in the mortality risk of subjects with high UACR compared to that with low UACR (HR 1.112, 95% CI 0.826–1.496, P = 0.486). In the high UNAPCR group, there was no significant difference in the mortality risks of subjects with high UACR, compared to that with low UACR (HR 0.805, 95% CI 0.545–1.191; P = 0.278). Because cancer history is a significant predictor of mortality and UNAPCR is associated with cancer history, multivariable Cox regression analyses were performed in the patients with cancer history and without cancer history. The results in either group were similar to that in the total population, except the association between the increased mortality risk and UNAPCR ≥ 120 mg/g did not reach a statistical significance in patients with cancer history and UACR ≥ 30 mg/g (HR 1.388, 95% CI 0.908–2.122; P = 0.130; Additional file 1: Table S5–S7).

**Table 2:** Cox regression analysis for the association between risk factors and all-cause mortality.

	Univariate model		Multivariate model	
	HR (95%CI)	*p* value	HR (95%CI)	*p* value
Groups classified by UACR & UNACR levels				
UACR < 30 mg/g & UNAPCR < 120 mg/g	ref		ref	
UACR ≥ 30 mg/g & UNAPCR < 120 mg/g	1.142 (0.857–1.522)	0.365	1.189 (0.889–1.589)	0.243
UACR < 30 mg/g & UNAPCR ≥ 120 mg/g	2.106 (1.395–3.180)	< 0.001	2.204 (1.448–3.356)	< 0.001
UACR ≥ 30 mg/g & UNAPCR ≥ 120 mg/g	2.179 (1.780–2.666)	< 0.001	1.796 (1.451–2.221)	< 0.001
Sex (male vs. female)	1.064 (0.912–1.242)	0.431		
Age (year)	1.062 (1.055–1.070)	< 0.001	1.058 (1.049–1.066)	< 0.001
Hypertension (yes vs. no)	2.338 (1.580–3.459)	< 0.001	0.729 (0.449–1.185)	0.202
CVD (yes vs. no)	2.282 (1.711–3.043)	< 0.001	1.345 (0.949–1.906)	0.094
Cancer (yes vs. no)	1.811 (1.555–2.109)	< 0.001	1.428 (1.222–1.670)	< 0.001
Glomerular disease (yes vs. no)	1.648 (1.372–1.979)	< 0.001	1.442(1.193–1.742)	< 0.001
TIN (yes vs. no)	1.091 (0.845–1.408)	0.506		
BMI ≥ 24 (kg/m^2^)	0.628 (0.538–0.733)	< 0.001	0.803 (0.686–0.940)	0.006
SBP ≥ 130 (mmHg)	1.026 (0.881–1.194)	0.740		
HbA1c (%)	1.069 (1.027–1.112)	0.001	1.088 (1.044–1.133)	< 0.001
CKD* (yes vs. no)	2.180 (1.829–2.599)	< 0.001	1.443 (1.195–1.742)	< 0.001
Low HDL cholesterol**	1.030 (0.886–1.196)	0.703		
LDL cholesterol ≥ 2.59 (mmol/L)	0.993 (0.854–1.155)	0.925		
Triglycerides ≥ 1.7 (mmol/L)	0.853 (0.730–0.996)	0.045	0.952 (0.811–1.118)	0.552
GPT (U/L)	0.996 (0.993–1.000)	0.049	1.002 (0.999–1.005)	0.170
Hb (g/L)	0.825 (0.796–0.855)	< 0.001	0.892 (0.856–0.929)	< 0.001
Statin use (yes vs. no)	0.765 (0.659–0.887)	< 0.001	0.806 (0.690–0.942)	0.007
SGLT2 inhibitor use (yes vs. no)	0.691 (0.172–2.770)	0.602		
GLP–1 RA use (yes vs. no)	0.934 (0.418–2.087)	0.868		
DPP–4 inhibitor use (yes vs. no)	0.798 (0.685–0.928)	0.003	0.777 (0.666–0.906)	0.001
RAS blockade use (yes vs. no)	1.282 (1.101–1.492)	0.001	1.189 (1.013–1.395)	0.034
Insulin use (yes vs. no)	1.405 (1.200–1.644)	< 0.001	1.384 (1.169–1.638)	< 0.001

*CKD was defined as eGFR < 60 mL/min/1.73 m^2^; **Low HDL cholesterol was defined as < 40 mg/dL (1.0 mmol/L) in men or < 50 mg/dL (1.3 mmol/L) in women. Variables with *P* value < 0.05 in the univariate Cox regression analysis were entered into the multivariate Cox regression analysis.

*BMI* body mass index, *CKD* chronic kidney disease, *CVD* cardiovascular disease, *DPP-4* dipeptidyl peptidase-4, *eGFR* estimated glomerular filtration rate, *GLP-1 RA* glucagon-like peptide-1 receptor agonist, *GPT* glutamate pyruvate transaminase, *Hb* hemoglobin, *HbA1c* hemoglobin A1c, *HDL* high-density lipoprotein , *LDL* low-density lipoprotein, *RAS* renin-angiotensin system, *SBP* systolic blood pressure, *SGLT2* sodium–glucose cotransporter 2, *TIN* Tubulointerstitial nephritis, *UACR* urine albumin-to-creatinine ratio, *UNAPCR* urine non-albumin protein-creatinine ratio.

**Table 3:** Analyses for association of UACR (or UNAPCR) levels with all-cause mortality using Cox regression analyses stratified by UNAPCR (or UACR) levels.

Stratum	Independent variable	HR (95% CI)	*p* value
UACR			
< 30 mg/g	UNAPCR (≥ 120 versus < 120 mg/g)	2.292 (1.440–3.649)	< 0.001
≥ 30 mg/g	UNAPCR (≥ 120 versus < 120 mg/g)	1.521 (1.188–1.947)	0.001
UNAPCR			
< 120 mg/g	UACR (≥ 30 versus < 30 mg/g)	1.112 (0.826–1.496)	0.486
≥ 120 mg/g	UACR (≥ 30 versus < 30 mg/g)	0.805 (0.545–1.191)	0.278

HRs were adjusted for age, hypertension, cardiovascular disease, cancer, glomerular disease, body mass index, hemoglobin A1c, estimated glomerular filtration rate, triglyceride, glutamate pyruvate transaminase, hemoglobin, statins use, dipeptidyl peptidase-4 inhibitors use, renin-angiotensin system blockade use, insulin use. *HR* hazard ratio, *UACR* urine albumin-to-creatinine ratio, *UNAPCR* urine non-albumin protein-creatinine ratio.

Table 4 shows the Cox regression for all-cause mortality in the multivariable model with the inclusion of both UACR and UNAPCR. UNAPCR ≥ 120 mg/g remained significantly associated with a higher risk of mortality (HR 1.655, 95% CI 1.324–2.070; P < 0.001), but UACR ≥ 30 mg/g was not significantly associated with the risk of mortality (HR 1.046, 95% CI 0.820–1.334; P = 0.717). The *p*-value for the interaction effect between UACR and UNAPCR regarding all-cause mortality was 0.126.

**Table 4:** Multivariate Cox regression analysis for all-cause mortality associated with UACR and UNAPCR mutually adjusted.

	HR (95%CI)	*p value*
UACR ≥ 30 mg/g	1.046 (0.820–1.334)	0.717
UNAPCR ≥ 120 mg/g	1.655 (1.324–2.070)	< 0.001
Age (year)	1.058 (1.049–1.066)	< 0.001
Hypertension (yes vs. no)	0.744 (0.458–1.208)	0.231
CVD (yes vs. no)	1.333 (0.940–1.890)	0.106
Cancer (yes vs. no)	1.431 (1.224–1.674)	< 0.001
Glomerular disease (yes vs. no)	1.439 (1.191–1.739)	< 0.001
BMI ≥ 24 (kg/m^2^)	0.801 (0.684–0.938)	0.006
HbA1c (%)	1.088 (1.044–1.133)	< 0.001
CKD* (yes vs. no)	1.426 (1.182–1.720)	< 0.001
Triglycerides ≥ 1.7 (mmol/L)	0.956 (0.815–1.123)	0.587
GPT (U/L)	1.002 (0.999–1.005)	0.175
Hb (g/L)	0.891 (0.855–0.929)	< 0.001
Statin use (yes vs. no)	0.811 (0.694–0.947)	0.008
DPP-4 inhibitor use (yes vs. no)	0.782 (0.671–0.913)	0.002
RAS blockade use (yes vs. no)	1.189 (1.014–1.395)	0.033
Insulin use (yes vs. no)	1.372 (1.160–1.624)	< 0.001

*P* value for interaction effect between UACR and UNAPCR on all-cause mortality was 0.126. *CKD was defined as eGFR < 60 mL/min/1.73 m^2^. Variables with *p* value < 0.05 in the univariate Cox regression analysis in Table 2 were entered into the multivariate Cox regression analysis.

*BMI* body mass index, *CKD* chronic kidney disease, *CVD* cardiovascular disease, *DPP-4* dipeptidyl peptidase-4, *eGFR* estimated glomerular filtration rate, *GPT* glutamate pyruvate transaminase, *Hb* hemoglobin, *HbA1c* hemoglobin A1c, *RAS* renin-angiotensin system, *UACR* urine albumin-to-creatinine ratio, *UNAPCR* urine non-albumin protein-creatinine ratio.

## Discussion

The main finding of our study indicates that UNAPCR is an independent predictor of all-cause mortality in patients with type 2 DM. Particularly, an increased UNAPCR was significantly predictive of mortality in patients without increased albuminuria, but increased albuminuria was not significantly associated with mortality in patients without increased UNAPCR. Although UACR was significantly associated with mortality in Cox regression analyses without adjusting for UNAPCR in the present study, this effect of UACR might result from a concordance between UACR and UNAPCR in the majority of the subjects (81.8%) and a positive correlation between UACR and UNAPCR levels. In line with our finding, the positive correlations were previously reported between UACR and UNAPCR levels^24,30^.

Albuminuria typically is indicative of glomerular damage^19^. Low-molecular-weight proteins that freely filtrate across the glomerulus but cannot be reabsorbed in the proximal tubule are regarded as tubular injury or protein overload due to excess protein production^31^. Non-albumin proteinuria may indicate tubulointerstitial injury-dominant kidney disease or other non-renal systemic diseases associated with the overproduction of low-molecular-weight proteins, such as immunoglobulin light chains in plasma cell dyscrasias^32^. Non-albumin proteinuria has been reported to be associated with tubulointerstitial damage according to renal biopsies^20,33^, and tubulointerstitial lesions progressed in parallel with glomerular lesions^34^. In patients with type 2 DM and biopsy-proven nephropathy, the tubulointerstitial lesion as assessed by interstitial fibrosis and tubular atrophy score has been identified as an independent predictor of renal events and all-cause mortality^35^. Therefore, non-albumin proteinuria may play an essential role beyond albuminuria in the pathological process associated with increasing mortality in patients with type 2 DM.

Various urinary non-albumin proteins have been previously reported in patients with diabetic nephropathy^36^. Studies have reported the prognostic value of non-albumin markers in terms of mortality in patients with DM, but these results were inconsistent. Urinary liver-type fatty acid-binding protein (L-FABP), an intracellular carrier protein of free fatty acids expressed predominantly on the proximal tubule, was revealed to be a predictor of all-cause mortality, independent of albuminuria in patients with DM^37,38^. However, Fufaa et al.^39^ found no significant association between urinary L-FABP and mortality. The kidney injury molecule-1 (KIM-1) to creatinine ratio, a marker of tubular injury, was found to be associated with mortality independent of albuminuria in patients with type 2 DM^40^. However, other studies have shown that the association between the ratio of KIM-1 to creatinine and mortality is not significant after adjustment for UACR in patients with type 2 DM^39,41^. Urinary concentrations of neutrophil gelatinase-associated lipocalin (u-NGAL), a tubular marker, have been reported to be associated with mortality risk^39,42^. However, in patients with type 2 DM and microalbuminuria, u-NGAL is not independently associated with all-cause mortality^43^. Although some evidence supports the potential utility of urinary non-albumin biomarkers for the risk prediction of mortality, measuring a specific tubular biomarker is still not cost-effective in clinical practice. In contrast, UNAPCR from spot urine samples is easily measured at outpatient clinics with low costs. UNAPCR may have the potential to be a useful marker because non-albumin proteinuria correlates well with urinary tubular markers, including L-FABP, KIM-1, and NGAL^44^.

Non-albumin proteinuria has been reported as an independent predictor of DKD progression and vascular complications in patients with type 2 DM^22,23,45^. However, the study exploring the prognostic value of non-albumin proteinuria on mortality was limited and the study focusing on patients with type 2 DM was unavailable. Chang et al.^25^ investigated the predictive values of UACR, UPCR, and UNAPCR on all-cause mortality in a population with 32.1% patients with DM and in the 3 × 3 risk matrices demonstrating HRs for all-cause mortality categorized by UPCR (< 150, ≥ 150 to < 500, and ≥ 500 mg/g) and UACR (< 30, ≥ 30 to < 300, and ≥ 300 mg/g). Interestingly, subjects with UACR < 30 and UPCR ≥ 500 mg/g had the highest risk of mortality among all groups compared to subjects with UACR < 30 and UPCR < 150 mg/g as the reference group in a multivariable model. In line with our findings, Chang et al. concluded that when compared with UPCR or UACR, UNAPCR had the most linear-appearing dose–response relationship with all-cause mortality.

Albuminuria has been reported as a risk factor for predicting CVD, DKD progression, and all-cause mortality in patients with type 2 DM^6,7^. Treatment with RAS blockade and SGLT2 inhibitors results in renal protection and mortality prevention, and these treatments are recommended for patients with increased UACR^46–50^. However, several studies have reported that DKD may also progress without an increased albuminuria^10–13,15^. Increasing mortality risk was also revealed in normoalbuminuric renal insufficiency in patients with DM^17,18^. A study based on data from the National Health and Nutrition Examination Surveys (NHANES) 1988–2006 reported that mortality rates generally trended downward over time for adults with DM and a UACR ≥ 30 mg/g. The increases in the use of RAS blockades among adults with DM and albuminuria during the past two decades may explain this finding. In contrast, mortality rates increased over time in those with eGFR < 60 mL/min/1.73 m^2^ and a UACR < 30 mg/g^16^. However, guidance of early assessing mortality risk or treatment for patients with this normoalbuminuric phenotype is still limited. In the present study, UNAPCR ≥ 120 mg/g and eGFR < 60 mL/min/1.73 m^2^ but not UACR were found to be independent predictors of all-cause mortality. Even though patients presented normoalbuminuria (UACR < 30 mg/g), the mortality risk still increased if patients concurrently had UNAPCR ≥ 120 mg/g and the increase of mortality risk in the group with UACR < 30 mg/g and UNAPCR ≥ 120 mg/g was similar to the increase in mortality risk in the group with UACR ≥ 30 mg/g and UNAPCR ≥ 120 mg/g. These findings suggest that using UACR alone to predict mortality and defining UACR < 30 mg/g as normality may underestimate the mortality risk in a relatively small subgroup with low UACR but increased UNAPCR. UNAPCR may have the potential to identify targeted patients with increasing mortality risk even in patients with UACR < 30 mg/g and type 2 DM.

### Limitations and strengths

The present study had several limitations. First, the enrolled participants were from a relatively homogeneous population, which was mono-ethic and single-center based, indicating that the results may not be generalizable to all populations with type 2 DM. Second, the present study enrolled patients in the diabetes P4P program, which has been reported to attenuate chronic diabetic complications^51–53^. Because we only enrolled patients with both UACR and UPCR data, which were routinely screened in the P4P programs for CKD in Taichung VGH, the prevalence of increased UACR might be higher in the present study than that previously reported^54^. Third, only a single spot urine sample was collected from each participant, which might introduce bias due to misclassification for proteinuria, because some physiological factors, such as exercise within 24 h and menstruation, might temporally affect urinary protein levels^8^. Fourth, residual confounding could not be completely avoided because information regarding patients’ lifestyles and environmental exposures was incomplete in the present study. Fifth, cancer and immune disorders might also be the causes of increased non-albumin proteinuria^55,56^. Despite the exclusion of patients with immune disorders from the study cohort and adjustments for the effects of cancer in the multivariable Cox regression analyses, cancer and immune disorders could only be identified based on ICD codes. Similarly, the effect of hematuria, which was not routinely screened in these P4P programs, could not be excluded. These conditions might have been underestimated, thus biasing the study results. Finally, this study was a retrospective cohort study; thus, we could not control other factors and treatments that patients received during the follow-up period. The strength of the present study is the indication of the role of UNAPCR as an independent predictor of all-cause mortality in patients with type 2 DM.

## Conclusions

In the present study, UNAPCR was found to be an independent predictor of all-cause mortality in patients with type 2 DM during a median follow-up period of 6.4 years after adjusting for the established markers including UACR and eGFR. Large-scale and multicenter prospective cohort studies are needed in the future to confirm these findings.

### Supplementary Information

Supplementary Information.

## Data Availability

The datasets used and/or analyzed during the current study are available from the corresponding author on reasonable request.

## Abbreviations

ANCA: Anti-neutrophil cytoplasmic autoantibody
CVD: Cardiovascular disease
CI: Confidence interval
CKD: Chronic kidney disease
DBP: Diastolic blood pressure
DKD: Diabetic kidney disease
DM: Diabetes mellitus
ESRD: End-stage renal disease
GLP-1 RA: Glucagon-like peptide-1 receptor agonist
GPT: Glutamic pyruvic transaminase
Hb: Hemoglobulin
HbA1c: Glycated Hemoglobulin
HDL: High-density lipoprotein
HR: Hazard ratio
ICD: Diagnostic international classification of diseases
KIM-1: Kidney injury molecule-1
LDL: Low-density lipoprotein
L-FABP: Liver-type fatty acid–binding protein
MCP-1: Monocyte chemoattractant protein-1
P4P: Pay-for-payment
SBP: Systolic blood pressure
SGLT2: Sodium–glucose cotransporter 2
TG: Triglycerides
UACR: Urinary albumin-to-creatinine ratio
UNAPCR: Urinary non-albumin protein-creatinine ratio
u-NGAL: Urinary concentrations of neutrophil gelatinase-associated lipocalin
UPCR: Urine protein-to-creatinine ratio
