# Supplementary material for: Urinary non-albumin protein-creatinine ratio is an independent predictor of mortality in patients with type 2 diabetes: a retrospective cohort study
Source: Sci Rep. 2024 May 8;14:10526. doi: 10.1038/s41598-024-61395-7 (PMC11078930; doi:10.1038/s41598-024-61395-7)

Table S1. The ICD codes used in this study.

| No. |  | ICD9-CM CODE | ICD10 CODE |
| --- | --- | --- | --- |
| 1 | Diabetes | 【250】【648】 | 【E08】-【E13】 |
| 2 | Type 1 diabetes | 【250.23】【250.31】  【250.33】【250.41】  【250.43】【250.53】  【250.61】【250.73】  【250.81】【250.93】 | 【E10】 |
| 3 | Other types of diabetes | 【251.8】 | 【E08】【E09】【E13】 |
| 4 | Gestational diabetes | 【648】 | 【O24】 |
| 5 | Hypertension | 【401】-【405】 | 【I10】-【I15】 |
| 6 | Cardiovascular disease –  Coronary arterial disease | 【410】-【414】 | 【I20】-【I25】 |
| 7 | Cardiovascular disease –  Cerebral vascular disease | 【430】-【438】 | 【I60】-【I69】 |
| 8 | Cardiovascular disease –  Peripheral arterial disease | 【443】 | 【I73】 |
| 9 | Autoimmune disease –  Systemic lupus erythematosus | 【710.0】 | 【**M32.8**】【**M32.9**】 |
| 10 | Autoimmune disease –  Rheumatoid arthritis | 【714.0】-【714.81】 | 【M05】【M06】 【M08】 |
| 11 | Autoimmune disease –  Sicca syndrome | 【710.2】 | 【M35.00】 【M35.01】 【M35.09】 |
| 12 | Autoimmune disease –  Anti-neutrophil cytoplasmic autoantibody vasculitis |  | 【I77.82】 |
| 13 | Cancer | 【140】-【208】 | 【C00】-【C97】 |
| 14 | Glomerular disease | 【583.0】-【583.7】 【584】 | 【N01】-【N03】【N05】-【N08】 |
| 15 | Tubulointerstitial nephritis | 【590】 | 【N10】-【N16】 |

*ICD* Diagnostic International Classification of Diseases.

Table S2.  Characteristics of all subjects, grouped by UACR of 30 mg/g or UNAPCR of 120 mg/g, respectively.

|  | Total  (n = 1809) |  | UACR  < 30 mg/g  (n = 529) | | UACR  ≥ 30 mg/g  (n = 1280) | *p* value |  | UNAPCR  < 120 mg/g  (n = 724) | UNAPCR  ≥ 120 mg/g  (n = 1085) | *p* value |
| --- | --- | --- | --- | --- | --- | --- | --- | --- | --- | --- |
| Age (year) | 69 ± 12 |  | | 69 ± 12 | 69 ± 13 | 0.891 |  | 68 ± 12 | 69 ± 12 | 0.198 |
| Female, n (%) | 677 (37.4%) |  | | 192 (36.3%) | 485 (37.9%) | 0.523 |  | 227 (31.4%) | 450 (41.5%) | <0.001 |
| UACR (mg/g) | 767.3 ± 1532.1 |  | | 11.9 ± 7.9 | 1079.9 ± 1728.1 | < 0.001 |  | 51.8 ± 164.6 | 1245.2 ± 1824.5 | <0.001 |
| UNAPCR (mg/g) | 561.7 ± 1308.9 |  | | 120.8 ± 521.5 | 744.2 ± 1482.2 | < 0.001 |  | 77.3 ± 24.4 | 885.4 ± 1611.4 | <0.001 |
| BMI (kg/m^2^) | 23.9 ± 3.9 |  | | 23.9 ± 3.7 | 23.9 ± 4.0 | 0.851 |  | 24.2 ± 3.7 | 23.7 ± 4.0 | 0.001 |
| SBP (mmHg) | 134 ± 19 |  | | 128 ± 17 | 137 ± 19 | < 0.001 |  | 130 ± 17 | 137 ± 20 | <0.001 |
| DBP (mmHg) | 74 ± 12 |  | | 73 ± 12 | 75 ± 12 | < 0.001 |  | 73 ± 12 | 74 ± 12 | 0.029 |
| HbA1c (%) | 7.5 ± 1.7 |  | | 7.2 ± 1.6 | 7.6 ± 1.8 | < 0.001 |  | 7.2 ± 1.5 | 7.7 ± 1.8 | 0.000 |
| Glucose (mmol/L) | 8.4 ± 4.7 |  | | 8.1 ± 4.9 | 8.5 ± 4.6 | 0.003 |  | 8.0 ± 4.5 | 8.6 ± 4.8 | 0.006 |
| eGFR (mL/min/1.73 m^2^) | 54.9 ± 32.6 |  | | 68.1 ± 31.4 | 49.5 ± 31.5 | < 0.001 |  | 66.2 ± 28.5 | 47.4 ± 32.9 | <0.001 |
| Total cholesterol (mmol/L) | 4.3 ± 1.1 |  | | 4.2 ± 1.0 | 4.3 ± 1.2 | 0.174 |  | 4.2 ± 0.9 | 4.4 ± 1.2 | 0.013 |
| HDL cholesterol (mmol/L) | 1.2 ± 0.4 |  | | 1.3 ± 0.4 | 1.2 ± 0.4 | 0.001 |  | 1.2 ± 0.4 | 1.2 ± 0.4 | 0.254 |
| LDL cholesterol (mmol/L) | 2.5 ± 0.9 |  | | 2.4 ± 0.8 | 2.5 ± 0.9 | 0.109 |  | 2.4 ± 0.7 | 2.6 ± 1.0 | 0.028 |
| Triglycerides (mmol/L) | 1.8 ± 1.6 |  | | 1.7 ± 1.4 | 1.9 ± 1.6 | < 0.001 |  | 1.8 ± 1.7 | 1.9 ± 1.5 | <0.001 |
| GPT (U/L) | 27.6 ± 25.7 |  | | 29.9 ± 28.5 | 26.6 ± 24.4 | < 0.001 |  | 30.1 ± 28.5 | 25.9 ± 23.5 | <0.001 |
| Hb (g/L) | 121.9 ± 21.1 |  | | 129.2 ± 18.5 | 118.9 ± 21.4 | < 0.001 |  | 129.7 ± 18.5 | 116.7 ± 21.2 | <0.001 |
| Disease, n (%) |  |  | |  |  |  |  |  |  |  |
| Hypertension, n (%) | 1671 (92.4%) |  | | 458 (86.6%) | 1213 (94.8%) | < 0.001 |  | 645 (89.1%) | 1026 (94.6%) | <0.001 |
| CVD, n (%) | 1568 (86.7%) |  | | 442 (83.6%) | 1126 (88.0%) | 0.012 |  | 612 (84.5%) | 956 (88.1%) | 0.028 |
| Cancer, n (%) | 538(29.7%) |  | | 164 (31.0%) | 374 (29.2%) | 0.450 |  | 197 (27.2%) | 341 (31.4%) | 0.054 |
| Glomerular disease, n (%) | 294 (16.3%) |  | | 63 (11.9%) | 231 (18.0%) | 0.001 |  | 90 (12.4%) | 204 (18.8%) | <0.001 |
| TIN, n (%) | 154 (8.5%) |  | | 40 (7.6%) | 114 (8.9%) | 0.351 |  | 58 (8.0%) | 96 (8.8%) | 0.532 |
| Drug use, n (%) |  |  | |  |  |  |  |  |  |  |
| OHA use, n (%) | 1443 (79.8%) |  | | 437 (82.6%) | 1006 (78.6%) | 0.053 |  | 592 (81.8%) | 851 (78.4%) | 0.084 |
| Statin use, n (%) | 1058 (58.5%) |  | | 297 (56.1%) | 761 (59.5%) | 0.194 |  | 421 (58.1%) | 637 (58.7%) | 0.813 |
| Fibrate use, n (%) | 118 (6.5%) |  | | 30 (5.7%) | 88 (6.9%) | 0.346 |  | 51 (7.0%) | 67 (6.2%) | 0.463 |
| SGLT2 Inhibitor use, n (%) | 9 (0.5%) |  | | 6 (1.1%) | 3 (0.2%) | 0.013 |  | 6 (0.8%) | 3 (0.3%) | 0.102 |
| GLP-1 RA use, n (%) | 19 (1.1%) |  | | 4 (0.8%) | 15 (1.2%) | 0.430 |  | 7 (1.0%) | 12 (1.1%) | 0.776 |
| DPP-4 Inhibitor use, n (%) | 849 (46.9%) |  | | 231 (43.7%) | 618 (48.3%) | 0.074 |  | 316 (43.6%) | 533 (49.1%) | 0.022 |
| RAS blockade use, n (%) | 968 (53.5%) |  | | 253 (47.8%) | 715 (55.9%) | 0.002 |  | 380 (52.5%) | 588 (54.2%) | 0.476 |
| Hypertensive drug use, n (%) | 1384 (76.5%) |  | | 353 (66.7%) | 1031 (80.5%) | < 0.001 |  | 519 (71.7%) | 865 (79.7%) | <0.001 |
| Insulin use, n (%) | 506 (28.0%) |  | | 101 (19.1%) | 405 (31.6%) | < 0.001 |  | 148 (20.4%) | 358 (33.0%) | <0.001 |
| Incidence of mortality  (deaths/100 person-years) | 6.4 |  | | 4.3 | 7.3 | < 0.001 |  | 4.1 | 8.2 | <0.001 |

Continuous data are expressed as means ± standard deviations, and categorical data are expressed as numbers (percentages).

*BMI* body mass index, *CVD* cardiovascular disease, *DBP* diastolic blood pressure, *DPP-4* dipeptidyl peptidase-4, *eGFR* estimated glomerular filtration rate, *GLP-1 RA* glucagon-like peptide-1 receptor agonist, *GPT* glutamate pyruvate transaminase, *Hb* hemoglobin, *HbA1c* hemoglobin A1c, *HDL* high-density lipoprotein, *LDL* low-density lipoprotein, *OHA* oral hypoglycemic agent, *RAS* renin-angiotensin system, *SBP* systolic blood pressure, *SGLT2* sodium–glucose cotransporter 2, *TIN* Tubulointerstitial nephritis, *UACR* urine albumin-to-creatinine ratio, *UNAPCR* urine non-albumin protein-creatinine ratio.

| Table S3. Cox regression analysis for all-cause mortality associated with UACR of 30 mg/g and UNAPCR of 120 mg/g, respectively. | | | | | | | | | |
| --- | --- | --- | --- | --- | --- | --- | --- | --- | --- |
|  | Univariable model | |  | Multivariable model for UACR | |  | Multivariable model for UNAPCR | |  |
|  | HR (95%CI) | *p* value |  | HR (95%CI) | *p* value |  | HR (95%CI) | *p* value |  |
| UACR ≥ 30 mg/g | 1.736 (1.446–2.084) | <0.001 |  | 1.464 (1.210–1.772) | <0.001 |  |  |  |  |
| UNAPCR ≥ 120mg/g | 2.068 (1.752–2.441) | <0.001 |  |  |  |  | 1.699 (1.425–2.024) | <0.001 |  |
| Sex (male vs. female) | 1.064 (0.912–1.242) | .431 |  |  |  |  |  |  |  |
| Age (year) | 1.062 (1.055–1.070) | <0.001 |  | 1.057 (1.049–1.065) | <0.001 |  | 1.058 (1.049–1.066) | <0.001 |  |
| Hypertension (yes vs. no) | 2.338 (1.580–3.459) | <0.001 |  | 0.763 (0.470–1.240) | 0.275 |  | 0.748 (0.461–1.213) | 0.239 |  |
| CVD (yes vs. no) | 2.282 (1.711–3.043) | <0.001 |  | 1.312 (0.925–1.862) | 0.128 |  | 1.330 (0.939–1.885) | 0.109 |  |
| Cancer (yes vs. no) | 1.811 (1.555–2.109) | <0.001 |  | 1.456 (1.246–1.702) | <0.001 |  | 1.429 (1.222–1.671) | <0.001 |  |
| Glomerular disease (yes vs. no) | 1.648 (1.372–1.979) | <0.001 |  | 1.446 (1.197–1.748) | <0.001 |  | 1.440 (1.192–1.740) | <0.001 |  |
| TIN (yes vs. no) | 1.091 (0.845–1.408) | .506 |  |  |  |  |  |  |  |
| BMI ≥ 24 (kg/m^2^) | 0.628 (0.538–0.733) | <0.001 |  | 0.794 (0.678–0.930) | 0.004 |  | 0.802 (0.685–0.939) | 0.006 |  |
| SBP ≥ 130 (mmHg) | 1.026 (0.881–1.194) | .740 |  |  |  |  |  |  |  |
| HbA1c (%) | 1.069 (1.027–1.112) | .001 |  | 1.097 (1.053–1.142) | <0.001 |  | 1.088 (1.044–1.133) | <0.001 |  |
| CKD* (yes vs. no) | 2.180 (1.829–2.599) | <0.001 |  | 1.443 (1.196–1.740) | <0.001 |  | 1.427 (1.184–1.722) | <0.001 |  |
| Low HDL cholesterol** | 1.030 (0.886–1.196) | .703 |  |  |  |  |  |  |  |
| LDL cholesterol ≥ 2.59 (mmol/L) | 0.993 (0.854–1.155) | .925 |  |  |  |  |  |  |  |
| Triglycerides ≥ 1.7 (mmol/L) | 0.853 (0.730–0.996) | .045 |  | 0.969 (0.825–1.137) | 0.698 |  | 0.957 (0.815–1.123) | 0.588 |  |
| GPT (U/L) | 0.996 (0.993–1.000) | .049 |  | 1.002 (0.999–1.004) | 0.308 |  | 1.002 (0.999–1.005) | 0.170 |  |
| Hb (g/L) | 0.825 (0.796–0.855) | <0.001 |  | 0.879 (0.844–0.916) | <0.001 |  | 0.891 (0.855–0.928) | <0.001 |  |
| Statin use (yes vs. no) | 0.765 (0.659–0.887) | <0.001 |  | 0.813 (0.696–0.950) | 0.009 |  | 0.810 (0.693–0.946) | 0.008 |  |
| SGLT2 Inhibitor use (yes vs. no) | 0.691 (0.172–2.770) | .602 |  |  |  |  |  |  |  |
| GLP-1 RA use (yes vs. no) | 0.934 (0.418–2.087) | .868 |  |  |  |  |  |  |  |
| DPP-4 Inhibitor use (yes vs. no) | 0.798 (0.685–0.928) | .003 |  | 0.795 (0.682–0.926) | 0.003 |  | 0.782 (0.671–0.912) | 0.002 |  |
| RAS blockade use (yes vs. no) | 1.282 (1.101–1.492) | .001 |  | 1.165 (0.994–1.367) | 0.060 |  | 1.191 (1.016–1.397) | 0.032 |  |
| Insulin use (yes vs. no) | 1.405 (1.200–1.644) | <0.001 |  | 1.398 (1.183–1.653) | <0.001 |  | 1.375 (1.163–1.626) | <0.001 |  |

*CKD was defined as eGFR < 60 ml/min/1.73m^2^; **Low HDL cholesterol was defined as < 40 mg/dl (1.0 mmol/L) in men or < 50 mg/dl (1.3 mmol/L) in women. Variables with *P* value < 0.05 in the univariable Cox regression analysis were entered into the multivariable Cox regression analysis.

*BMI* body mass index, *CKD* chronic kidney disease, *CVD* cardiovascular disease, *DPP-4* dipeptidyl peptidase-4, *eGFR* estimated glomerular filtration rate, *GLP-1* *RA* glucagon-like peptide-1 receptor agonist, *GPT* glutamate pyruvate transaminase, *Hb* hemoglobin, *HbA1c* hemoglobin A1c, *HDL* high-density lipoprotein, *LDL* low-density lipoprotein, *RAS* renin-angiotensin system, *SBP* systolic blood pressure, *SGLT2* sodium–glucose cotransporter 2, *TIN* Tubulointerstitial nephritis, *UACR* urine albumin-to-creatinine ratio, *UNAPCR* urine non-albumin protein-creatinine ratio.

Table S4. Cox regression analysis for the association between all-cause mortality and groups classified by quartiles of UNAPCR.

|  | Univariable model | |  | Multivariable model | |
| --- | --- | --- | --- | --- | --- |
|  | HR (95%CI) | *p* value |  | HR (95%CI) | *p* value |
| Groups classified by quartiles of UNAPCR |  |  |  |  |  |
| Q1 (<88.1 mg/g) | ref. |  |  | ref. |  |
| Q2 (88.2 to 158.4 mg/g) | 2.116 (1.650–2.713) | <0.001 |  | 1.636 (1.272–2.105) | <0.001 |
| Q3 (158.5 to 415.9 mg/g) | 2.458 (1.924–3.140) | <0.001 |  | 1.816 (1.408–2.341) | <0.001 |
| Q4 (≥416.0 mg/g) | 3.156 (2.480–4.015) | <0.001 |  | 2.800 (2.152–3.643) | <0.001 |

HRs were adjusted for age, hypertension, cardiovascular disease, cancer, glomerular disease, body mass index, hemoglobin A1c, estimated glomerular filtration rate, triglyceride, glutamate pyruvate transaminase, hemoglobin, statins use, dipeptidyl peptidase-4 inhibitors use, renin-angiotensin system blockade use, insulin use. *HR* hazard ratio, *UNAPCR* urine non-albumin protein-creatinine ratio.

Table S5. Multivariable Cox regression analyses for the association between risk factors and all-cause mortality in the population with cancer history or without cancer history.

|  | with cancer history | |  | without cancer history | |
| --- | --- | --- | --- | --- | --- |
|  | HR (95%CI) | *p* value |  | HR (95%CI) | *p* value |
| Groups classified by UACR & UNACR levels |  |  |  |  |  |
| UACR < 30 mg/g & UNAPCR <120 mg/g | ref. |  |  | ref. |  |
| UACR ≥ 30 mg/g & UNAPCR <120 mg/g | 1.146 (0.715–1.839) | 0.571 |  | 1.274 (0.878–1.850) | 0.202 |
| UACR < 30 mg/g & UNAPCR ≥ 120 mg/g | 2.099 (1.129–3.905) | 0.019 |  | 2.316 (1.288–4.163) | 0.005 |
| UACR ≥ 30 mg/g & UNAPCR ≥ 120 mg/g | 1.621 (1.172–2.243) | 0.004 |  | 2.001 (1.507–2.657) | <0.001 |

HRs were adjusted for age, hypertension, cardiovascular disease, cancer, glomerular disease, body mass index, hemoglobin A1c, estimated glomerular filtration rate, triglyceride, glutamate pyruvate transaminase, hemoglobin, statins use, dipeptidyl peptidase-4 inhibitors use, renin-angiotensin system blockade use, insulin use. *HR* hazard ratio, *UACR* urine albumin-to-creatinine ratio, *UNAPCR* urine non-albumin protein-creatinine ratio.

Table S6. Analyses for association of UACR (or UNAPCR) levels with all-cause mortality using Cox regression analyses stratified by UNAPCR (or UACR) levels in the population with cancer history.

| Stratum |  | Independent variable | HR (95% CI) | *p* value |
| --- | --- | --- | --- | --- |
| UACR | <30 mg/g | UNAPCR (≥120 versus <120 mg/g) | 2.117 (1.330–3.499) | 0.046 |
|  | ≥30 mg/g | UNAPCR (≥120 versus <120 mg/g) | 1.388 (0.908–2.122) | 0.130 |
| UNAPCR | <120 mg/g | UACR (≥30 versus <30 mg/g) | 0.966 (0.583–1.603) | 0.894 |
|  | ≥120 mg/g | UACR (≥30 versus <30 mg/g) | 0.732 (0.412–1.300) | 0.287 |

HRs were adjusted for age, hypertension, cardiovascular disease, cancer, glomerular disease, body mass index, hemoglobin A1c, estimated glomerular filtration rate, triglyceride, glutamate pyruvate transaminase, hemoglobin, statins use, dipeptidyl peptidase-4 inhibitors use, renin-angiotensin system blockade use, insulin use. *HR* hazard ratio, *UACR* urine albumin-to-creatinine ratio, *UNAPCR* urine non-albumin protein-creatinine ratio.

Table S7. Analyses for association of UACR (or UNAPCR) levels with all-cause mortality using Cox regression analyses stratified by UNAPCR (or UACR) levels in the population without cancer history.

| Stratum |  | Independent variable | HR (95% CI) | *p* value |
| --- | --- | --- | --- | --- |
| UACR | <30 mg/g | UNAPCR (≥120 versus <120 mg/g) | 3.016 (1.583–5.747) | 0.001 |
|  | ≥30 mg/g | UNAPCR (≥120 versus <120 mg/g) | 1.562 (1.148–2.126) | 0.005 |
| UNAPCR | <120 mg/g | UACR (≥30 versus <30 mg/g) | 1.196 (0.820–1.745) | 0.353 |
|  | ≥120 mg/g | UACR (≥30 versus <30 mg/g) | 0.891 (0.514–1.543) | 0.680 |

HRs were adjusted for age, hypertension, cardiovascular disease, cancer, glomerular disease, body mass index, hemoglobin A1c, estimated glomerular filtration rate, triglyceride, glutamate pyruvate transaminase, hemoglobin, statins use, dipeptidyl peptidase-4 inhibitors use, renin-angiotensin system blockade use, insulin use. *HR* hazard ratio, *UACR* urine albumin-to-creatinine ratio, *UNAPCR* urine non-albumin protein-creatinine ratio.

**Fig. S1.** Cumulative survival estimate based on the Kaplan–Meier method. (A) Cumulative survival grouped by UACR of 30 mg/g. (B) Cumulative survival grouped by UNAPCR of 120 mg/g. *UACR,* urine albumin-to-creatinine ratio; *UNAPCR,* urine non-albumin protein-creatinine ratio.


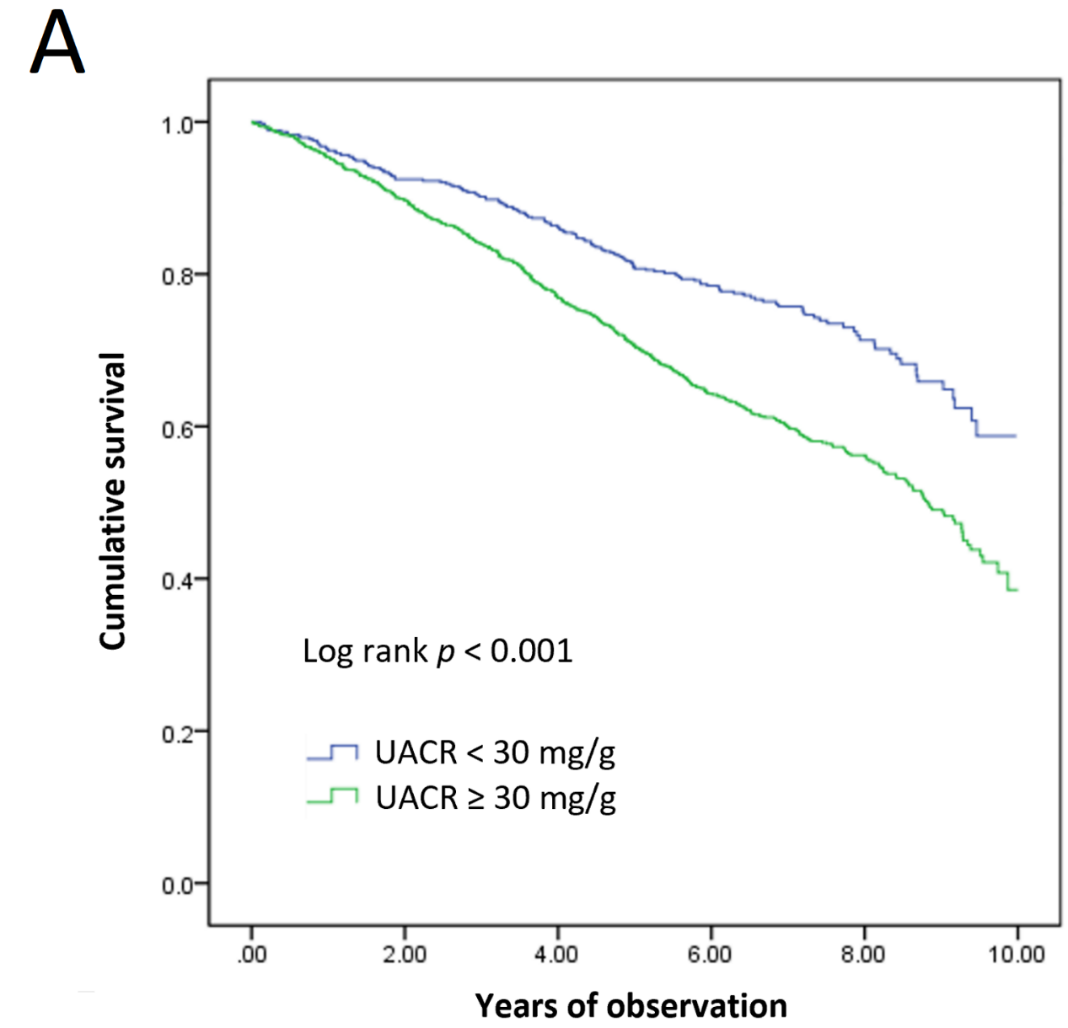

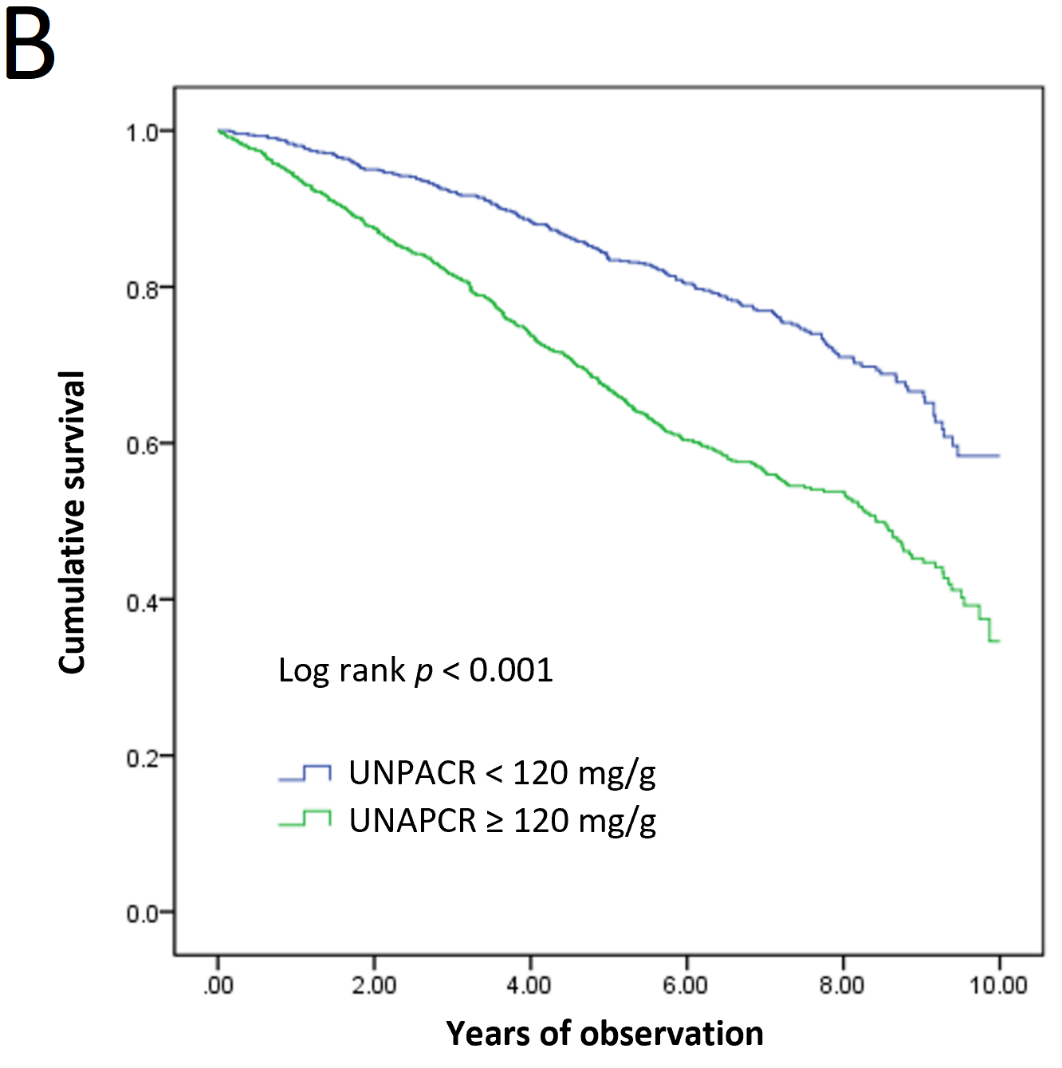


**Fig. S2.** Cumulative survival estimate based on the Kaplan–Meier method in four groups classified by quartiles of UNAPCR. *UNAPCR,* urine non-albumin protein-creatinine ratio.


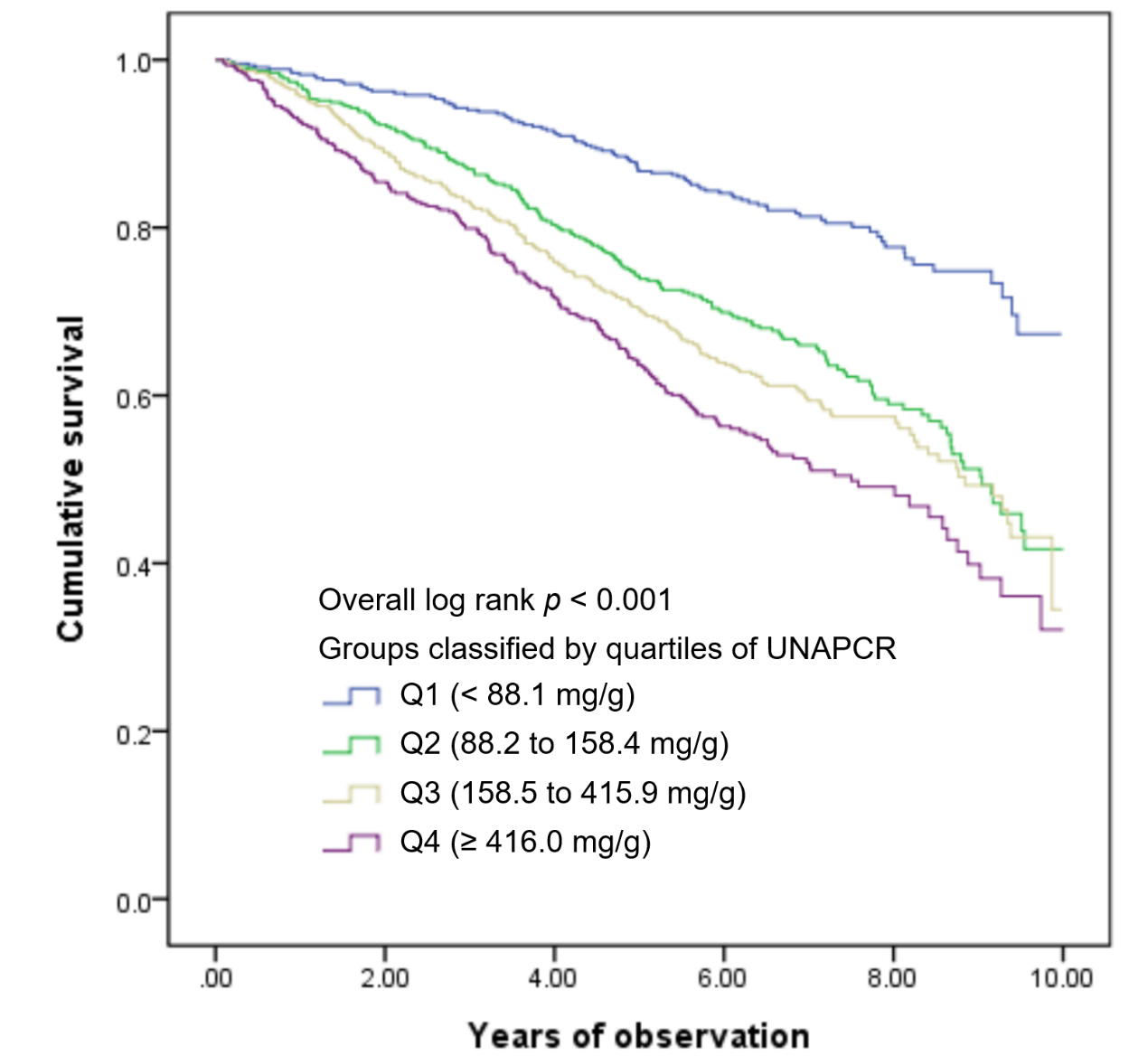

Supplement: Supplementary file 1 — Supplementary Information. [file 41598_2024_61395_MOESM1_ESM.docx]
